# Adherence to long-term use of renin-angiotensin II-aldosterone system inhibitors in children with chronic kidney disease

**DOI:** 10.1186/s12887-019-1434-6

**Published:** 2019-02-20

**Authors:** Chien-Ning Hsu, Shiou-Huei Huang, You-Lin Tain

**Affiliations:** 1 Department of Pharmacy, Kaohsiung Chang Gung Memorial Hospital, Kaohsiung, Taiwan; 2 School of Pharmacy, Kaohsiung Medical University, Kaohsiung, Taiwan; 3 Division of Pediatric Nephrology, Department of Pediatrics, Kaohsiung Chang Gung Memorial Hospital and Chang Gung University, College of Medicine, 123 Dabi Road, Niausung, Kaohsiung, 83301 Taiwan

**Keywords:** Medication adherence, Pediatrics, Chronic kidney disease, ACE inhibitor, Angiotensin receptor blocker

## Abstract

**Background:**

Although renin-angiotensin II-aldosterone system inhibitor (RASI) use for renal protection is well-documented, adherence to RASI therapy in the pediatric population is unclear. This study aimed to evaluate patient characteristics associated with adherence to chronic RASI use in patients with childhood chronic kidney disease (CKD).

**Methods:**

Childhood CKD was identified using ICD-9 codes in the population-based, Taiwan national health insurance research database between 1997 and 2011. Patients continuously receiving RASIs for ≥3 months without interruption > 30 days after CKD diagnosis were defined as incident users. Medication adherence was measured as the proportion of days covered (PDC) by RASI prescription refills during the study period. Multivariate logistic regression was employed to assess the odds for adherence (PDC ≥80%) to RASI refills.

**Results:**

Of 1271 incident users of RASI chronic therapy, 16.9% (*n* = 215) had PDC ≥80%. Compared to the group with PDC < 80%, patients in the high adherence group more often had proteinuria (aOR [adjusted odds ratio]1.93; 95%CI [confidence interval], 1.18–3.17), anemia (aOR, 1.76; 95% CI, 1.20–2.58), and time to start of chronic use > 2 years (aOR, 1.12; 95%CI, 1.06–1.19). Odds of being non-adherent were increased by hypertension and older ages (comparing to < 4 years) at start of chronic use, 9–12 years (aOR, 0.38; 95%CI, 0.17–0.82), 13–17 years (aOR, 0.45; 95%CI, 0.22–0.93),≥18 years (aOR, 0.34; 95%CI 0.16–0.72) and males (aOR, 0.68; 95%CI, 0.49–0.94).

**Conclusions:**

The rate of RASI prescription refilling adherence was relatively low and associated with CKD-specific comorbid conditions. This study identifies factors associated with low adherence and highlights the need to identify those who should be targeted for intervention to achieve better blood pressure control, preventing CKD progression.

**Electronic supplementary material:**

The online version of this article (10.1186/s12887-019-1434-6) contains supplementary material, which is available to authorized users.

## Background

Childhood chronic kidney disease (CKD) increases the risk of renal replacement therapy, cardiovascular disease, and premature death in the pediatric population. Randomized controlled trials have shown that renin-angiotensin II-aldosterone system inhibitors (RASIs), including angiotensin-converting enzyme inhibitors (ACEI) and angiotensin II receptor blockers (ARB), may control blood pressure [1], reduce proteinuria [2, 3], and slow CKD progression to end-stage renal disease (ESRD) in the pediatric population [4]. However, the effect of recommendations for the use of and adherence to RASI therapy in routine pediatric care settings remain unclear.

Medication adherence refers to the degree to which patients take their medications as prescribed (e.g., once daily), as well as whether they continue to take a prescribed medication [5]. Non-adherence is a growing concern to professionals, healthcare systems, and other stakeholders (e.g., payers) because of mounting evidence showing that 33 to 80% of youth are reportedly non-adherent to their prescribed chronic medicines [6–8], resulting in poorer outcomes and higher costs of care in children and adolescents with chronic illness [9]. Assessment of pediatric patient medication adherence and use of interventions to improve adherence are limited in routine practice. Although the reasons for non-adherence vary, identifying patients by their adherence to a specific medication can facilitate effective intervention for the patients most likely to benefit [10].

Medication adherence in children and adolescents has been examined for childhood CKD, but limited in a short term (recent 7 days) time frame [11, 12]. Medication therapy is complex and a major burden for pediatric patients and their parents. Moreover, pediatric patients with progressive CKD require multiple classes of medications to delay progression (e.g., corticosteroids, immunosuppressive agents) and prevent and/or treat comorbid conditions (e.g., anti-hypertensives, phosphate binders, and lipid and iron medications). Considering that medication burden may be associated with poor adherence, as in adults [13], it is critical to understand patient-related factors and disease conditions related to long-term RASI use in order to improve adherence in children and adolescents with CKD who require multiple therapies. The aim of this study was to investigate adherence to ACEI/ARB/aliskiren initiation and concomitant medication use in children and adolescents with CKD. In addition, patient demographic and clinical factors associated with adherence to ACEI/ARB were identified.

## Methods

### Data sources and study sample

A population-based cohort study was conducted using the National Health Insurance Research Database (NHIRD), which includes 99% of the 23 million persons enrolled in the Taiwan National Health Insurance (NHI) program [14]. Briefly, Taiwan’s NHI program is a government-run, single-payer, compulsory program implemented on March 1, 1995; details on the universal, comprehensive coverage are described elsewhere [15, 16]. De-identified information in the NHIRD includes date of birth, sex, area of residence, diagnostic codes, prescriptions, and medical procedures. The study was approved by the institutional review board at Chang Gung Medical Foundation at Taoyun, and informed consent was waived due to use of de-identified personal information in the NHIRD.

Following Kidney Disease: Improving Global Outcomes (KDIGO) guidelines for diagnosis of childhood CKD, the International Classification of Diseases, Ninth Revision (ICD-9) was used to identify individuals who had CKD diagnoses on at least 2 occasions (Additional file 1: Table S1) within 1 year, at least 90 days apart, from January 1, 2000, through December 31, 2011. Patients who died or commenced renal replacement therapy before the date of CKD diagnosis or who met the criteria for CKD at age ≥ 20 years were also excluded. Ultimately, 51,846 patients with childhood-onset CKD were selected for the current study.

### RAS inhibitors assessment

Patients who had taken any ACEI/ARB/aliskiren after the index date were defined as new RASI users. All analyses were conducted on an as-treated basis according to the chronic use of RASI therapy for at least 90 days, with a permissible gap of less than 30 days during the follow-up period. The start date of continuous chronic therapy was defined as the RASI index date.

Using pharmacy prescription refill data, adherence was assessed by the proportion of days covered (PDC), which is defined as the number of follow-up days covered with medication, divided by the total number of days in follow-up. The PDC truncates any oversupply during a specific observation period and is widely used in health care setting as a tool to measure health care quality [17]. The PDC boundary is between 0 and 1, and represents the proportion of days with a prescription for RASI, as determined by the date the prescription was filled and the days supplied. PDC was evaluated as the mean overall and subgroup PDC, stratified by adherence (≥0.80 and < 0.80), and based on the correlation with cardiovascular outcomes in adult populations [18].

### Patient characteristics

Demographics, including gender and age were assessed on the index date. Baseline CKD-related comorbid conditions [19], including diabetes mellitus, hypertension-related diseases and conditions, hyperlipidemia, proteinuria, mineral bone disorder (MBD), and anemia, were defined by using ICD-9 codes or medications used for at least 3 months for a particular disease within 1 year before the RASI index date. A detailed drug classification is shown in Additional file 1: Table S2. Pill burden was assessed according to the number of pharmacological classes in which prescribed for ≥28 days by a 6-month interval between 1 year before the RASI index date and follow up. Time since CKD diagnosis to the RASI index date was assessed to determine early or late chronic use.

### Statistical analysis

Demographic and clinical characteristics were reported using the median (interquartile range [IQR]) for continuous variables and the frequency (percentage) for categorical variables. Patients with PDC ≥80 and < 80% in the study period were compared with respect to baseline characteristics, groups of medications used, and comorbid conditions. A multivariate logistic regression analysis was conducted to assess baseline patient and medication-related factors (i.e., age, sex, previous comorbid conditions, and the number of medication groups) associated with high adherence (PDC ≥80%). Two-sided *p* values less than 0.05 were considered statistically significant. Operationalized definitions of all diagnosis, procedure, and medication codes are included in Additional file 1: Tables S1 and S2. All analyses were conducted using SAS 9.3 (SAS Institute, Cary, NC, USA).

## Results

### Characteristics of the study cohort

**Fig. 1:**
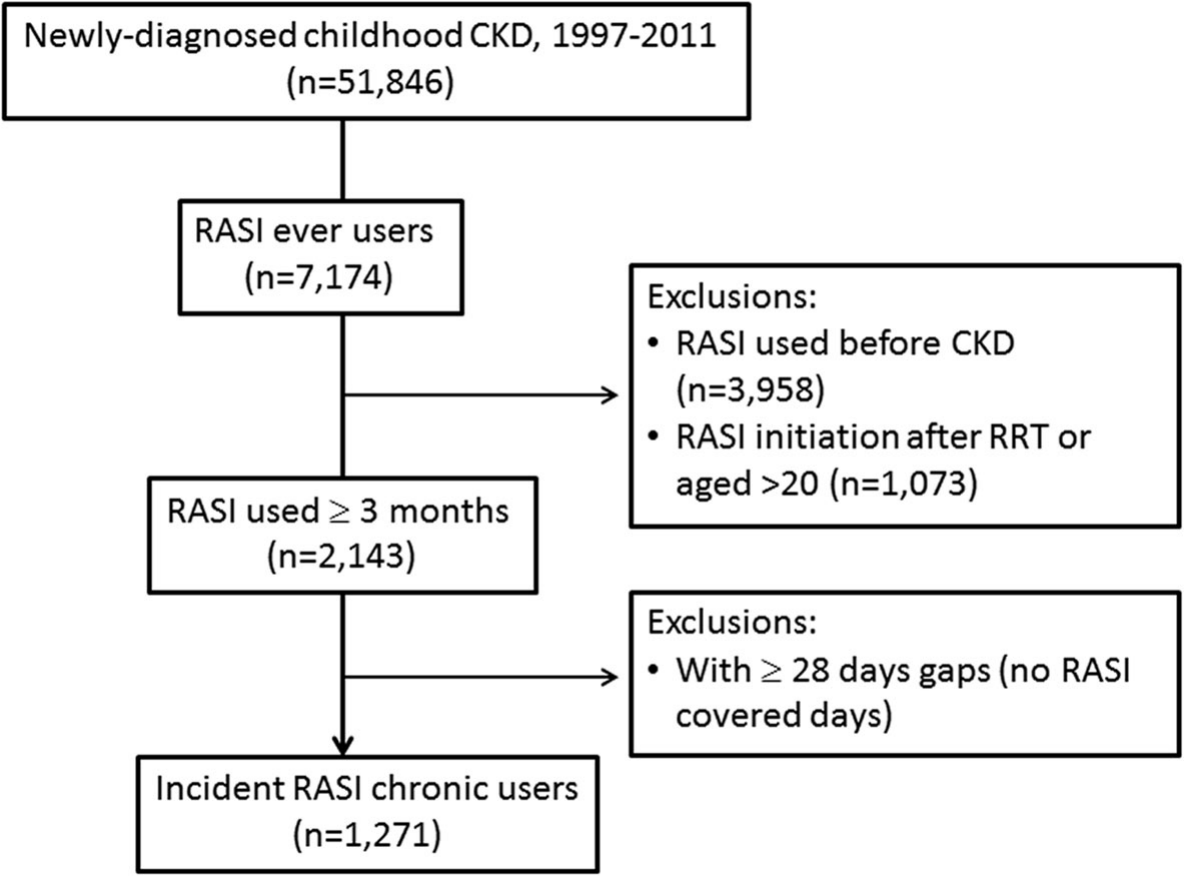
Patient selection process

Of the 51,846 children diagnosed as having CKD, 7174 (13.84%) children who were ever prescribed a RASI and 1271 other children met inclusion and exclusion criteria for chronic use (Fig. 1). The majority of patients were diagnosed with glomerular disease at baseline (68%), proteinuria (15%), hematuria (11.64%), and nephritis (10.54%). The mean age (standard deviation) of the cohort was 14.39 (4.86) years old, with 67% of patients over 13 years old (Table 1). Hypertension-related comorbid conditions (98.19%) and proteinuria (78.76%) were the most prevalent baseline comorbidities, and patients with PDC ≥80% more often had proteinuria (87.44% versus 76.99%) and anemia (26.05% versus 13.83%) than those with PDC < 80%. Time from CKD diagnosis to RASI index date was approximately 2 years (median 1.79, 25th–75th percentile, 0.74–3.71). Within 1 year prior to the RASI index date, the majority of the study cohort had been treated with antihypertensive therapy. A RASI was the most popular option (84.82%), as over 50% of treated patients had proteinuria (Table 2).

**Table 1:** Patient characteristics grouped by PDC

	Overall (*n* = 1271)		PDC < 80 (*n* = 1056)		PDC ≥ 80 (*n* = 215)		*P* value
Age at RASI index date, mean (SD), year	14.39 (4.86)		14.40 (4.79)		14.35 (5.19)		0.513
< 4	58	4.56	45	4.26	13	6.05	
5–8	148	11.64	120	11.36	28	13.02	
9–12	209	16.44	180	17.05	29	13.49	
13–17	522	41.07	431	40.81	91	42.33	
≥ 18	334	26.28	280	26.52	54	25.12	
Sex, n, %							0.003
Female	621	48.86	496	46.97	125	58.14	
Male	650	51.14	560	53.03	90	41.86	
CKD diagnosis, n, %							
CAKUT	114	8.97	95	9.00	19	8.84	0.941
Glomerular diagnosis	859	67.58	699	66.19	160	74.42	0.020
Diabetes/hypertension/gout- related nephropathy	17	1.34	14	1.33	3	1.40	1.000
Nephrotic syndrome	310	24.39	275	26.04	35	16.28	0.002
Glomerulonephritis	301	23.68	267	25.28	34	15.81	0.003
Systemic lupus erythematous	308	24.23	206	19.51	102	47.44	<.0001
Others	488	38.39	430	40.72	58	26.98	<.0001
Nephritis	134	10.54	115	10.89	19	8.84	0.464
Bartter syndrome/Fabry disease	4	0.31	3	0.28	1	0.47	0.524
Proteinuria	191	15.03	166	15.72	25	11.63	0.143
Hematuria	148	11.64	131	12.41	17	7.91	0.062
CKD	65	5.11	57	5.40	8	3.72	0.396
miscellaneous	27	2.12	23	2.18	4	1.86	1.000
Baseline comorbid conditions, n, %							
Proteinuria	1001	78.76	813	76.99	188	87.44	<.000
Anemia	202	15.89	146	13.83	56	26.05	<.000
HTN-related	1248	98.19	1040	98.48	208	96.74	0.092
Mineral bone disorders	103	8.10	83	7.86	20	9.30	0.493
Diabetes	100	7.87	86	8.14	14	6.51	0.488
Hyperlipidemia	273	21.48	219	20.74	54	25.12	0.172
Time to RASI chronic therapy, year							
mean (SD)	2.67 (2.55)		2.55 (2.46)		3.26 (2.89)		<.000
median (IQR)	1.79 (0.74–3.71)		1.68 (0.72–3.54)		2.38 (1.07–4.57)		

*PDC* proportion of days covered, *CKD* chronic kidney disease, *CAKUT* congenital anomalies of kidney and urinary tract, *HTN* hypertension, *RASI* renin-angiotensin II-aldosterone system inhibitor, *IQR* interquartile range (25th- 75th percentile)

**Table 2:** Prior medications used for existing hypertension and proteinuria

Concomitant medications	Overall (n = 1271)		PDC < 80 (n = 1056)		PDC ≥ 80 (n = 215)	
Hypertension, n, %	1114	87.65	912	86.36	202	93.95
C02 (antihypertensive)	24	1.89	12	1.14	12	5.58
C03 (diuretics)	193	15.18	153	14.49	40	18.60
C04 (vasodilators)	94	7.40	78	7.39	16	7.44
C07 (beta-blockers)	61	4.80	40	3.79	21	9.77
C08C (dihydropyridines)	130	10.23	90	8.52	40	18.60
C08D (non-dihydropyridines)	10	0.79	6	0.57	4	1.86
C09 (RASI)	1078	84.82	882	83.52	196	91.16
Proteinuria, n, %	679	53.42	525	49.72	154	71.63
Corticosteroids	660	51.93	510	48.30	150	69.77
L04 (immunosuppressants)	247	19.43	170	16.10	77	35.81
L01 (anti-neoplastic agents)	131	10.31	97	9.19	34	15.81
P02 (mycophenolate)	40	3.15	34	3.22	6	2.79

*PDC* proportion of days covered during study follow-up. Concomitant medication use was categorized using ATC codes for the sum of 90 days of supply within one year prior to the RASI index date

**Fig. 2:**
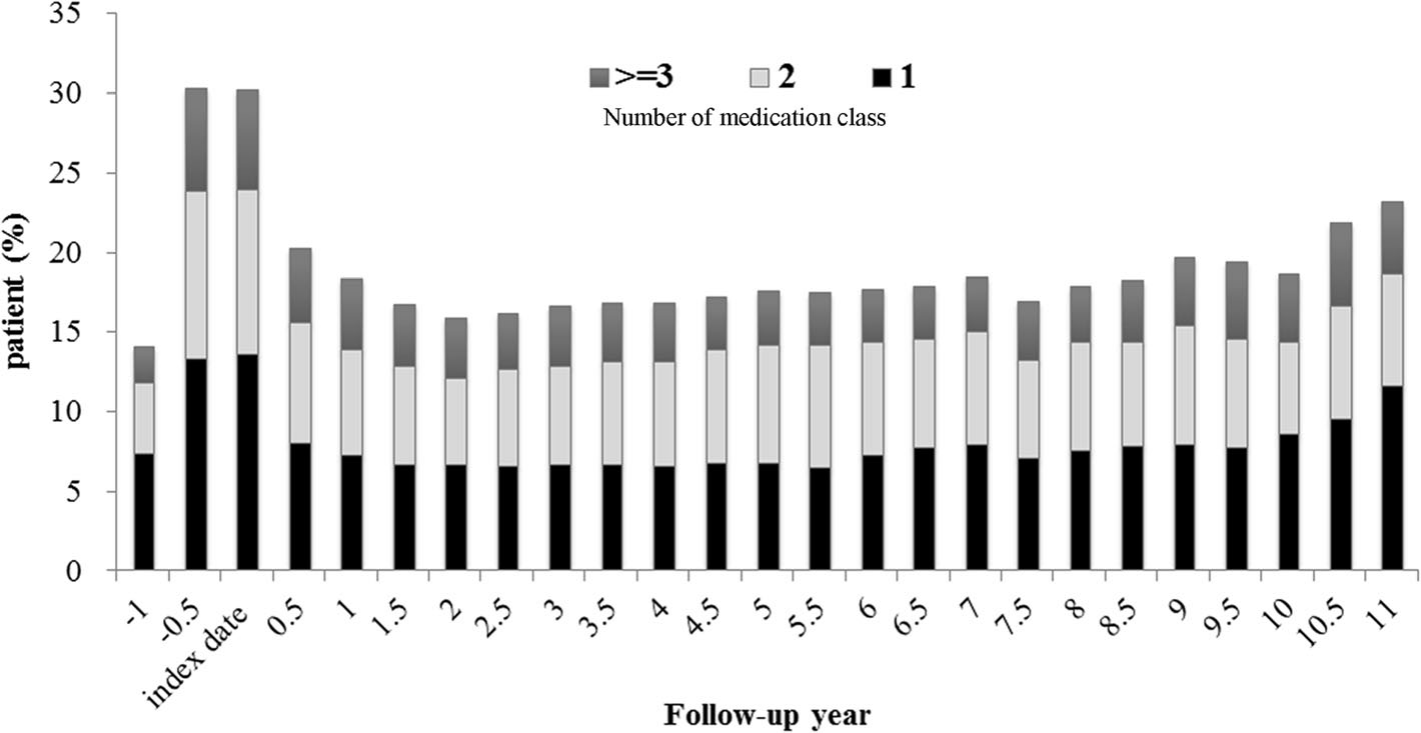
Trend in number of selected medication class per person among RASI chronic users

**Fig. 3:**
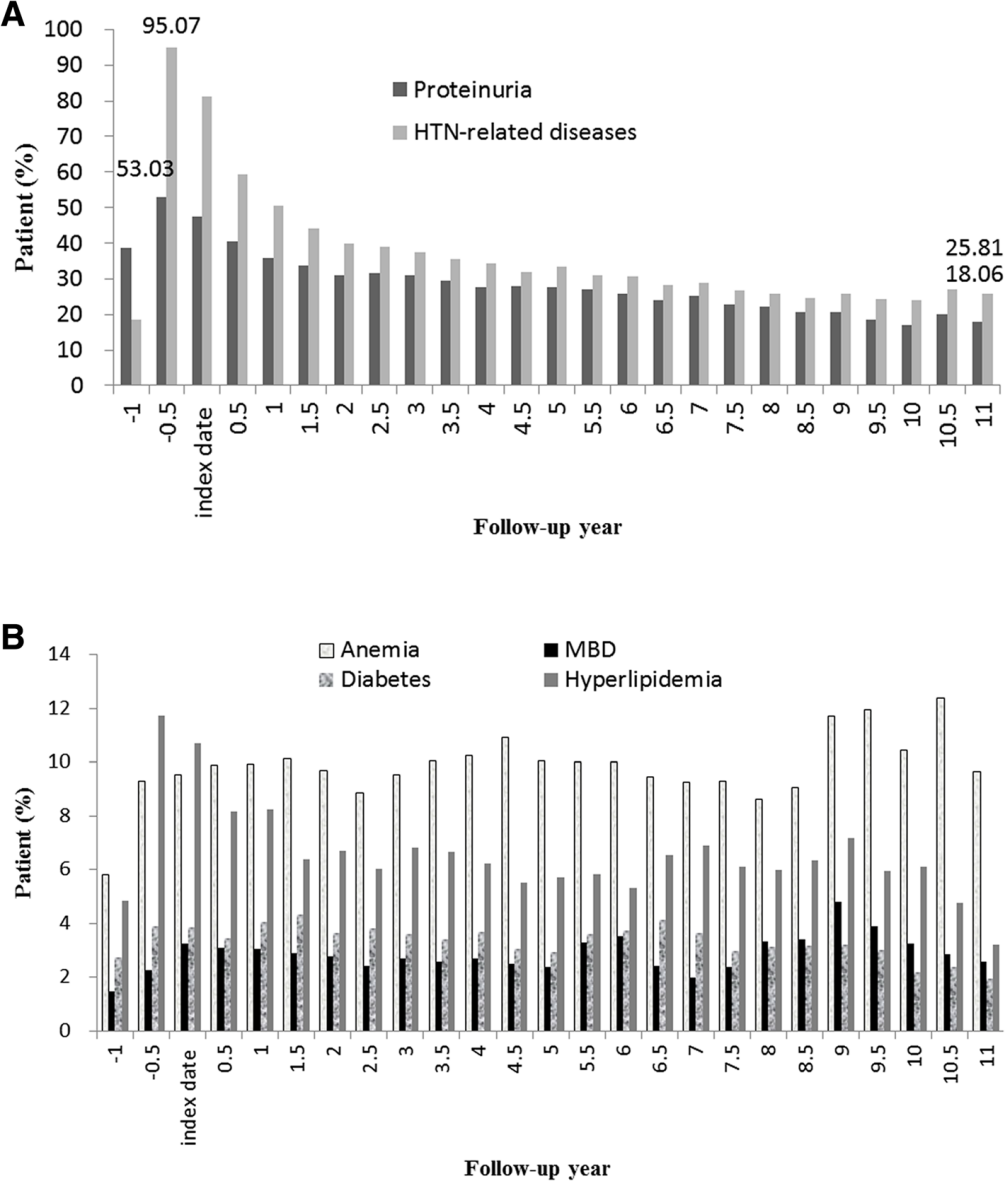
Trend in use of individual medication class among RASI chronic users. **a** Medication classes for proteinuria, hypertension-related diseases. **b** Medication classes for anemia, mineral bone disorders, diabetes and hyperlipidemia

For the targeted comorbid conditions, the trends in pill burden (number of medication classes) varied over the entire study period. The percentage of patients using ≥3 classes of medications increased from the RASI index period (including the prior and post 6 months covering the index date), slowly decreased to 3.29% at 5.5 years, and then gradually increased to 5.24% at 10.5 years, during the 11 years of follow-up (Fig. 2). There was a declining trend in the rate of medication use for hypertension-related diseases (from 81.37 to 25.81%) and proteinuria (from 47.3 to 18.06%); on the other hand, the rates of medication use for anemia (lowest-highest, 9.05–12.38%), hyperlipidemia (3.23–10.7%), mineral bone disorders (2–4.79%) and diabetes (2–4.32%) were low but remained steady during follow-up (Fig. 3A and B).

### Factors associated with RASI non-adherence

In multivariate analysis including baseline patient and clinical characteristics (Table 3), 3 factors associated with increased odds of being adherent to chronic RASI use included proteinuria (adjusted odds ratio [aOR]: 1.93; 95% confidence interval [CI]: 1.18–3.17; *p* = 0.010), anemia (aOR 1.76; 95% CI 1.20–2.58; *p* = 0.004), and late RASI initiation (aOR 1.12; 95% CI 1.06–1.19; *p* < 0.001). On the other hand, patient age 9–12 years (aOR 0.38; 95% CI 0.17–0.82; *p* = 0.014), 13–17 years (aOR 0.45; 95% CI 0.22–0.93; *p* = 0.031) and ≥ 18 years (aOR 0.34; 95% CI 0.16–0.72; *p* = 0.005), male (aOR 0.68; 95% CI 0.49–0.94; *p* = 0.018), and presence of hypertension-related disease at baseline (aOR 0.32; 95% CI 0.12–0.86; *p* = 0.023) were associated with decreased odds of being adherent to chronic RASI therapy. In the same model, older age at the start of chronic RASI therapy was associated with 20% less (aOR, 0.81 per 3-year increase; 95% CI0.70–0.94; *p* = 0.006) likely to be adherent.

**Table 3:** Estimated odds ratios for being associated with medication adherence to RASI chronic therapy

	OR	95% CI		*P* value
Age at index date, years^a^				
< 4	1			
5–8	0.65	(0.30	1.43)	0.288
9–12	0.38	(0.17	0.82)	0.014
13–17	0.45	(0.22	0.93)	0.031
≥ 18	0.34	(0.16	0.72)	0.005
Male gender	0.68	(0.49	0.94)	0.018
Comorbid conditions				
Proteinuria	1.93	(1.18	3.17)	0.010
Anemia	1.76	(1.20	2.58)	0.004
HTN-related	0.32	(0.12	0.86)	0.023
Mineral bone disorders	1.06	(0.60	1.88)	0.839
Diabetes	0.92	(0.48	1.75)	0.790
Hyperlipidemia	1.09	(0.75	1.59)	0.656
Number of ATCs group (initial < 6 months)	1.31	(0.42	4.08)	0.641
Time to RASI chronic therapy	1.12	(1.06	1.19)	<.001
CKD diagnosis				
CAKUT	1			
Glomerular diagnosis	1.19	(0.59	2.39)	0.626
Others	0.70	(0.33	1.48)	0.351
≥ 2 types of diagnosis	0.64	(0.29	1.42)	0.278

^a^The odd ratio was 0.81 per 3-year increase (95%CI, 0.70–0.94; p = 0.006) in the same regression model; ATC: Anatomical Therapeutic Chemical (ATC) classification coding system (Additional file 1: Table S2)

## Discussion

The study used a population-based claims database to investigate prescription patterns and adherence to RASI for childhood CKD. The findings revealed that overall adherence to chronic RASI therapy was low and was highly correlated with the presence of CKD-related comorbid conditions among children and adolescents with CKD. Adherence to RASI therapy can be partially explained by clinical factors, such as advanced aggressive comorbidities, time since CKD diagnosis, and patient age and gender. However, the time to adherence with chronic therapy was not fully supported by guidelines, suggesting that more research on childhood CKD is needed to increase medication adherence.

The rate of ACEI/ARB use in our study was consistent with previous registry and claims database studies on childhood CKD [4] or childhood hypertension [20]. Age at the start of chronic RASI therapy in the present study (14.39 ± 4.86 years old) was similar to other pediatric reports (range: 11–17 years old) in Western countries [1, 11, 21]. Children with RASI more frequently had proteinuria and hypertension and related complications [11].

However, our finding of a low rate of adherence to chronic RASI treatment in children with CKD concurs with few studies. ACEIs and ARBs have been shown to decrease systemic and glomerular pressure and reduce proteinuria more effectively than other antihypertensive medications [1]. In patients with mild or transient proteinuria, no treatment or a short-course of treatment may be necessary. Dynamic strategies of antihypertensive therapy involving switching, combination, or monotherapy may be used in the group of patients with uncontrolled or resistant hypertension. This may explain the higher rate of RASI use at baseline, but adherence to chronic therapy was low after CKD diagnosis in our study cohort.

Adherence to RASI therapy might be influenced by adverse drug reactions and tolerability concerns (e.g., skin rashes, angioedema, hyperkalemia), which may lead to treatment interruption. For example, patients treated with ACEIs or ARBs should be monitored for hypotension, early decrease in glomerular filtration rate, and hyperkalemia [22]. Although the KDIGO guidelines state that these common side effects usually can be managed without discontinuation of the agent [22], they may lead to non-adherence in actual practice. Thus, a low rate of chronic RASI use should be considered justification for referral to multiple pediatric specialists in a real-world setting. A review study suggests that physician specialty and familiarity with antihypertensive regimens play a significant role in the management of hypertension [23]. Other factors were associated with medication adherence. For example, RASI adherence was associated with the presence of anemia and proteinuria. This is consistent with previous reports [11, 21] and clinical experience; more progressive CKD and/or symptomatic medical conditions may enforce patient adherence to chronic medication therapy.

There is a paucity of research examining time to ACEI/ARB initiation and its impacts on treatment adherence and persistence in clinical practice. However, a cross-sectional study suggested that CKD duration had no effect on medication adherence, although only the prior 7 days of adherence were evaluated [11].

CKD and associated comorbidities impose a substantial pill burden on children and adolescents. It explains that a longer time since CKD diagnosis, which implies a more advanced stage of kidney disease, is more likely to be associated with adherence than a shorter duration of CKD in the present study. But, ACEI/ARB (18%) was one of the most frequently reported not being taken prescription (slightly lower than 23% for alkali treatments, 26% for phosphate binders and 25% growth hormone) in a childhood CKD cohort [11]. In that study, the number of medication classes was higher in children with advanced stage of CKD; however, neither a larger number of medicines used nor worsening estimated glomerular filtration rate (eGFR) was found to be independently associated with 7-day medication adherence in childhood CKD [11]. Not number of medication groups or types of CKD, but comorbidity is linked with RASI adherence were consistent with our findings. This effect could be explained by patient understanding of the importance of medication in association with progressive comorbidity and kidney function deterioration. Non-adherence to chronic medication was frequently reported among adolescents [11, 24] and males in other pediatric studies, similar to our study findings.

Growing evidence has shown that hypertension is undertreated in pediatric populations. For example, almost half of all children and adolescents with CKD had uncontrolled hypertension: 44.1% (*n* = 744) in multiple pediatric nephrology clinics in Taiwan [25], and 48.5% (*n* = 202) in a registered US pediatric cohort [26]. Children with hypertension not receiving RASI therapy have an increased prevalence of uncontrolled blood pressure [20, 26]. The delay to start of chronic RASI therapy may be a contributor to the high prevalence of cardiovascular disease and kidney function deterioration. Moreover, continuous users of RASI therapy had a superior renal protective effect, compared with non-users (37% lower) and short-term users (21% lower), with slowing of CKD progression in an observational registry trial [4].

Both updated European and US clinical guidelines emphasize the importance of diagnosis and management of hypertension in children with and without CKD, ACEI or ARB is recommended to treat CKD children with hypertension and proteinuria to reduce their risk of cardiovascular complications and CKD progression [27] There is a general paucity of both medication adherence and persistence evidence for children with CKD worldwide. This article represents the first step toward a better understanding of chronic medication adherence in a large childhood CKD cohort, comparable with other populations of pediatric patients with CKD in the literature. The implications for clinical practice given these findings are multiple: (1) reviewing patient’s medication history is the most useful way for clinicians to evaluate adherence; (2) adherence evaluation should occur at regular intervals in a practice setting, in order to identify possible medication-related problems (such as adverse drug reactions) that may interfere with adherence [28, 29], (3) effective interventions to improve adherence include educational interventions, providing specific information about CKD and its comorbidities, and prescribed treatment according to the adolescent’s cognitive abilities and health literacy, empowering adolescents to deal with adherence issues, and ensuring family support and motivational therapy [24]. The 80% threshold of PDC following the first 90 days RASI therapy may shed light on the optimal signal for the intervention of mediation nonadherence to discriminate poor outcome risk in childhood CKD.

This study was subject to certain limitations common to studies using claims data. First, laboratory results regarding proteinuria, hypertension, or disease severity (e.g., eGFR stage) are not available in the NHI dataset. This limitation was addressed by using the proxy of the number of medication classes at 3-month intervals. Second, medication adherence was determined by indirect measurement using pharmacy prescription filling data. Accurate medication adherence is difficult to examine, and is a challenge at both individual and population levels [30]. Pharmacy refill adherence for antihypertensive medication is superior to self-reporting to enable correlate with cardiovascular disease incidence in elderly populations [31]. Pharmacy refills are calculated using monthly data points over 3 months (i.e., as-treated effect) and are more reflective of adherence behavior over time, but may yield a more conservative, lower rate than other measures of adherence. Another limitation of this study is that the switching of RASIs to other categories of antihypertensive therapy and discontinuation due to adverse ACEI/ARB reactions were unclear, which limits the ability to draw conclusions about causes of non-adherence. Further research is needed to investigate patient and family member attitudes toward medication use for chronic illness, as well as barriers and challenges to adherence.

## Conclusions

Adherence to chronic RASI therapy in children and adolescents with CKD was heavily influenced by age and comorbid conditions related to progressive kidney disease in the present study cohort. Further research on understanding of the role of demographics (i.e., adolescents and young adults, males), the CKD care delivery process, and socioeconomic determinants of prescribing is imperative for the design of effective intervention strategies to improve management of hypertension and related chronic complications in childhood and young adulthood.

## Additional file

Additional file 1: **Table S1.** ICD 9 codes for disease conditions in the study. **Table S2.** Medications used to treat chronic kidney disease and its related comorbid conditions. (DOCX 21 kb)

## Abbreviations

ACEI/ARB: angiotensin-converting enzyme inhibitors/angiotensin II receptor blockers
aOR: adjusted odds ratio
ATC: Anatomical Therapeutic Chemical (ATC) classification coding system
CAKUT: congenital anomalies of kidney and urinary tract
CI: confidence interval
CKD: chronic kidney disease
eGFR: estimated glomerular filtration rate
HTN: hypertension
ICD-9: International Classification of Diseases, Ninth Revision
IQR: interquartile range
KDIGO: Kidney Disease: Improving Global Outcomes
MBD: mineral bone disorder
NHI: National Health Insurance
NHIRD: National Health Insurance Research Database
PDC: proportion of days covered
RASI: renin-angiotensin II-aldosterone system inhibitor
