# Supplementary material for: Adherence to long-term use of renin-angiotensin II-aldosterone system inhibitors in children with chronic kidney disease
Source: BMC Pediatr. 2019 Feb 20;19:64. doi: 10.1186/s12887-019-1434-6 (PMC6383266; doi:10.1186/s12887-019-1434-6)
Supplement: Supplementary file 1 — Table S1. ICD 9 codes for disease conditions in the study. Table S2. Medications used to treat chronic kidney disease and its related comorbid conditions. (DOCX 21 kb) [file 12887_2019_1434_MOESM1_ESM.docx]

**Table S1** ICD 9 codes for chronic kidney disease and comorbid conditions.

| Disease condition | ICD-9-CM Code |
| --- | --- |
| Chronic kidney disease | 2504, 2551, 2727, 2741, 28311, 4031, 40412, 40413, 40492, 40493, 4421, 4473,5724, 580, 581, 582, 583, 585, 586 (or V451), 587, 588, 5880,5881, 5888, 5889, 7885, 7910, 59654, 5997, 7100  CAKUT:  589, 591, 59371, 59372, 5960, 753: 7531, 7532, 753.20, 753.21, 753.22, 753.23, 753.29, 7530, 7532, 7533, 7534, 7536, 7538, 7539  Note: 1.Any two ICD-9 codes for CKD presented within 1 year, at least 90 days apart, from January 1, 2000, through December 31, 2011.  2. ICD9: 591 (hydronephrosis), 5997 (hematuria), 584 (acute renal failure) needs to present with other ICD9 codes for CKD within 1 year, at least 90 days apart. |
| Dialysis | Taiwan National Health Insurance program billing code:  Hemodialysis: 58001C, 58019C, 58020C, 58021C, 58022C, 58023C, 58024C, 58025C, 58027C, 58029C  Peritoneal dialysis: 58002C, 58011A, 58011B, 58011C, 58017A, 58017B, 58017C, 58026C, 58028C, 58011AB |
| Hypertension-related diseases and conditions | 401, 402, 403, 404, 405, 437.2, 7962 |
| Hyperlipidemia | 272, 4140, 429, 437 (excluding 4372), 440, 445 |
| Diabetes mellitus | 249, 250, 251, 90, V5867 |
| Anemia | 280-285 |
| Proteinuria | 7910 |
| Mineral and bone disorders | 2520, 588, 2682, 7906, 2753 |

ICD9 codes for each 6 targeted CKD-related comorbid conditions were identified within one year prior to the date of CKD diagnosis.

**Table S2** Medications used to treat chronic kidney disease and its related comorbid conditions.

| Drugs | ATC code |
| --- | --- |
| Hypertension | C02AC, C02CA, C02DB-DD, C02K, C02L, C02N,  C03A, C03B, C03CA, C03DA, C03E, C03X, C04A, C07, C08 CA, C08DA/C08DB, C09A/C09B, C09C/C09D,C09X |
| Proteinuria | H02AB, H0B, L04A |
| Anemia | B03A, B03B, B03XA |
| Diabetes | A10A, A10B |
| Mineral and bone disorders | V03AE, H05B, H05BX, A11CC |
| Lipid modifying agents | C10A, C10B |

Anatomical Therapeutic Chemical (ATC) classification coding system was used to identify and categorize the medication use for each specific disease and conditions.
